# Supplementary material for: Systematic characterization of Ustilago maydis sirtuins shows Sir2 as a modulator of pathogenic gene expression
Source: Front Microbiol. 2023 Apr 11;14:1157990. doi: 10.3389/fmicb.2023.1157990 (PMC10126416; doi:10.3389/fmicb.2023.1157990)
Supplement: Supplementary file 1 [file Table_1_v1.DOCX]

**TABLE S1. Strains used in this study.**

| **Strain** | **Relevant genotype** | **Reference** |
| --- | --- | --- |
| SG200 | *a1:mfa2 bW2bE1* | Bölker et al., 1995 |
| SG200 Δ*sir2* | *a1:mfa2 bW2bE1* Δ*sir2*::nat | This work |
| SG200 Δ*hst2* | *a1:mfa2 bW2bE1* Δ*hst2*::nat | This work |
| SG200 Δ*hst5* | *a1:mfa2 bW2bE1* Δ*hst5*::gen | This work |
| SG200 Δ*hst6* | *a1:mfa2 bW2bE1* Δ*hst5*::hyg | This work |
| SG200 *sir2*:eGFP | *a1:mfa2 bW2bE1* *sir2*:eGFP:hyg | This work |
| SG200 *hst2*:eGFP | *a1:mfa2 bW2bE1* *hst2*:eGFP:hyg | This work |
| SG200 *hst4*:eGFP | *a1:mfa2 bW2bE1* *hst4*:eGFP:hyg | This work |
| SG200 *hst5*:eGFP | *a1:mfa2 bW2bE1* *hst5*:eGFP:hyg | This work |
| SG200 *hst6*:eGFP | *a1:mfa2 bW2bE1* *hst6*:eGFP:hyg | This work |
| SG200 Δs*ir2* + *sir2* | *a1:mfa2 bW2bE1* *sir2*::nat::*sir2*:gen | This work |
| SG200 P*otef*:*sir2* | *a1:mfa2 bW2bE1* *ip*R[P*otef*:*sir2*]*ip*S | This work |
| SG200 P*pit2*:*sir2* | *a1*:*mfa2 bW2bE1* *ip*R[P*pit2*:*sir2*]*ip*S | This work |
| FBD11 | a1a2/b2b2 | Banuett and Herskowitz, 1989 |
| FBD11 Δ*hst4* | a1a2/b2b2 *hst4*/Δ*hst4*::gen | This work |

Banuett, F., and Herskowitz, I. (1989). Different a alleles of *Ustilago maydis* are necessary for maintenance of filamentous growth but not for meiosis. *Proc. Natl. Acad. Sci.* 86, 5878–5882. doi: 10.1073/pnas.86.15.5878.

Bölker, M., Böhnert, H. U., Braun, K. H., Görl, J., and Kahmann, R. (1995). Tagging pathogenicity genes in *Ustilago maydis* by restriction enzyme-mediated integration (REMI). *Mol. Gen. Genet. MGG* 248, 547. doi: 10.1007/BF02423450.
