# Supplementary material for: Systematic characterization of Ustilago maydis sirtuins shows Sir2 as a modulator of pathogenic gene expression
Source: Front Microbiol. 2023 Apr 11;14:1157990. doi: 10.3389/fmicb.2023.1157990 (PMC10126416; doi:10.3389/fmicb.2023.1157990)
Supplement: Supplementary file 2 [file Table_2_v2.docx]

**TABLE S2. Primers used in this study.**

| **Gene** | **Use** | **Name** | **Primer sequence (5´-3´)** |
| --- | --- | --- | --- |
| *sir2* | Deletion construct | sir2KO5_fwd | AAAGGTGCTACTCAACCTCGTCG |
|  |  | sir2KO5_rev | CACGGCCTGAGTGGCCGTAGCAATTCCAAGCCGAGACG |
|  |  | sir2KO3_fwd | GTGGCCATCTAGGCCGAGTACGACACCCAATGGAC |
|  |  | sir2KO3_rev | CGACGCAGCATATCCGAAGC |
|  | Deletion verification | sir2CO5_fwd | AGATGATGATGCCGATGCTACC |
|  |  | nat2_rev | TGTACGCATGTAACATTATACTGAAAACCT |
|  |  | nat1_fwd | TGGCTGCTGATCACAGCAAGTCAGATT |
|  |  | sir2CO3_rev | CATACACGAGCAACGGCAACG |
|  |  | sir2KOint_fwd | TGACAAGACCTTCTTTCTCTCC |
|  |  | sir2KOint_rev | AGTGGAGGATTGGCGTCTTGT |
|  | Complementation | sir2KO5_fwd | AAAGGTGCTACTCAACCTCGTCG |
|  |  | sir2EC5_rev | CACGGCCTGAGTGGCCTCTTCAACAGGAAGGGGAGC |
|  |  | sir2EC5_fwd | GTGGCCATCTAGGCCTCATAGATCACCTCTCTCTC |
|  |  | sir2EC3_rev | TCTCGACATCGGTGACAAGG |
|  | Complementation verification | sir2CO5_fwd | AGATGATGATGCCGATGCTACC |
|  |  | gen1_rev | TCTTCTGAGCGGGACTCTGG |
|  |  | gen2_fwd | GTACGGGTACATCGGATCTGC |
|  |  | sir2CO3_rev | CATACACGAGCAACGGCAACG |
|  |  | sir2KOint_fwd | TGACAAGACCTTCTTTCTCTCC |
|  |  | sir2KOint_rev | AGTGGAGGATTGGCGTCTTGT |
|  | eGFP tagging | sir2ORF_fwd | TCGAGGTCGACGGTATCGATAAGCTTGATAAAGACATGTTTGACAAGACC |
|  |  | sir2ORF_rev | GGTGAACAGCTCCTCGCCCTTGCTCACCATTGATGGAGCCTGTTGTGAATGC |
|  |  | eGFPHyg_sir2_fwd | ATGGTGAGCAAGGGCGAGGA |
|  |  | eGFPHyg_sir2_rev | ATAGGGCGAATTGGAGCTCG |
|  |  | sir2Ter_fwd | CCTGAGTGGCCGAGCTCCAATTCGCCCTATCATCTCCTTCGAGTACGACA |
|  |  | sir2Ter_rev | GCTCTAGAACTAGTGGATCCCCCGGGCTGCACAGCTTCATCCCGTACTGG |
|  |  | sir2GFP_fwd | AAGACATGTTTGACAAGACC |
|  |  | sir2GFP_rev | TGATGGAGCCTGTTGTGAATGC |
|  | Overexpression with P*otef* | sir2StartNcoI | TGCCATGGGCGGCAAAGCCTTCCAAAG |
|  |  | sir2StopNotI | CAGCGGCCGCTTATGATGGAGCCTGTTGTG |
|  | Overexpression with P*otef* | sir2StartSacII | TATCCGCGGATGGGCGGCAAAGCCTTCC |
|  |  | sir2StopXbaI | ATTCTAGATTATGATGGAGCCTGTTGTG |
|  | *ip* locus integration verification | N_Sdh2_fwd | TCCTGTCTTTTCGGCAAGACTCTTCG |
|  |  | N_pDL51_otef_rev | TGGTGCACTCTCAGTACAATCTGC |
|  |  | Amp1_fwd | TTCTGTGACTGGTGAGTACTCAACC |
|  |  | N_Sdh2_rev | TAAGTGACGATTGCGAGTTCTCTTGG |
| *hst2* | Deletion construct | hst2KO5_fwd | GCCATTGTTGTGTGTGTATGGATCG |
|  |  | hst2KO5_rev | CACGGCCTGAGTGGCCGGCATCCAATCCCAGAATACG |
|  |  | hst2KO3_fwd | GTGGCCATCTAGGCCTTTGGTCGTGGTGGTTGTGC |
|  |  | hst2KO3_rev | CGCATACGAGACAGAGACAGG |
|  | Deletion verification | hst2CO5_fwd | TACACCGGCATTGTTAATCAGC |
|  |  | nat2_rev | AGATGATGATGCCGATGCTACC |
|  |  | nat1_fwd | TGGCTGCTGATCACAGCAAGTCAGATT |
|  |  | hst2CO3_rev | GAAAGCTCATTTCTTCCCGTCG |
|  |  | hst2KOint_fwd | TGTATCCGGGCAACTTCAAGC |
|  |  | hst2KOint_rev | CCCATGACAATGAGTAGGTCG |
|  | eGFP tagging | hst2ORF_fwd | CGAATTCCTGCAGCCCGGGGATGCCAGAGACAAAGAGC |
|  |  | hst2ORF_rev | TGCTCACCATTGATGACGGTTTGTTCGAAAC |
|  |  | eGFPHyg_hst2_fwd | ACCGTCATCAATGGTGAGCAAGGGCGAG |
|  |  | eGFPHyg_ hst2_rev | CGGCGTCTCCTATTAATGCGGCCGCACAG |
|  |  | hst2Ter_fwd | CGCATTAATAGGAGACGCCGACATGCAG |
|  |  | hst2Ter_rev | CGGCCGCTCTAGAACTAGTGTGATCTGCTTTCCTCCTTATCCG |
|  |  | hst2GFP_fwd | ATGCCAGAGACAAAGAGC |
|  |  | hst2GFP_rev | TGATCTGCTTTCCTCCTTATCCG |
| *hst4* | Deletion construct | hst4KO5_fwd | AGGATGACAGACAATCCACC |
|  |  | hst4KO5_rev | CACGGCCTGAGTGGCCATGACTCGTGACGAAGTAGG |
|  |  | hst4KO3_fwd | GTGGCCATCTAGGCCTATGCATGATGTGTGTTCG |
|  |  | hst4KO3_rev | TGGGTAGGAAGGTAAGCAGG |
|  | Deletion verification | hst4CO5_fwd | TTCACAGAAAGAGGAAAAGC |
|  |  | gen1_rev | TCTTCTGAGCGGGACTCTGG |
|  |  | gen2_fwd | GTACGGGTACATCGGATCTGC |
|  |  | hst4CO3_rev | CTGGTTTCCAATCTGTCTGG |
|  | eGFP tagging | hst4ORF_fwd | CGAATTCCTGCAGCCCGGGGCCTGCTGAGGCCAAAGCC |
|  |  | hst4ORF_rev | TGCTCACCATAAGACAAGCAGCCGTCTC |
|  |  | eGFPHyg_hst4_fwd | TGCTTGTCTTATGGTGAGCAAGGGCGAG |
|  |  | eGFPHyg_ hst4_rev | CTATATGCGCTATTAATGCGGCCGCACAG |
|  |  | hst4Ter_fwd | CGCATTAATAGCGCATATAGTGCGGAATG |
|  |  | hst4Ter_rev | CGGCCGCTCTAGAACTAGTGGTGTCCTAAGTCGAAATTG |
|  |  | hst4GFP_fwd | CCTGCTGAGGCCAAAGCC |
|  |  | hst4GFP_rev | GTGTCCTAAGTCGAAATTGG |
| *hst5* | Deletion construct | hst5KO5_fwd | GTACTACTGCATGGTCAAGC |
|  |  | hst5KO5_rev | CACGGCCTGAGTGGCCCTAGCCAAGTGCACTCTTGC |
|  |  | hst5KO3_fwd | GTGGCCATCTAGGCCATAGAGGATGAAACCACGCG |
|  |  | hst5KO3_rev | ACCTTGTTGGTCTTTCTTGG |
|  | Deletion verification | hst5CO5_fwd | GCGAAGACGATGAAAATGG |
|  |  | gen1_rev | TCTTCTGAGCGGGACTCTGG |
|  |  | gen2_fwd | GTACGGGTACATCGGATCTGC |
|  |  | hst5CO3_rev | GAAGCTCAGCGAATCATGC |
|  |  | hst5KOint_fwd | AGAAGGCTACCGTAAAGACG |
|  |  | hst5KOint_rev | ACGATTCAGGACCATGACG |
|  | eGFP Tagging | hst5ORF_fwd | GATATCGAATTCCTGCAGCCCGGGGACATTATAGGCCGATCTTCTACCAC |
|  |  | hst5ORF_rev | CCTTGCTCACCATACTGCTGATCACGCCGCT |
|  |  | eGFPHyg_hst5_fwd | CGTGATCAGCAGTATGGTGAGCAAGGGCGAG |
|  |  | eGFPHyg_ hst5_rev | ATCCTCTATGACCTATTAATGCGGCCGCACAG |
|  |  | hst5Ter_fwd | GCCGCATTAATAGGTCATAGAGGATGAAACCACG |
|  |  | hst5Ter_rev | GGTGGCGGCCGCTCTAGAACTAGTGAGTCGCTCCACGCACTTC |
|  |  | hst5GFP_fwd | ACATTATAGGCCGATCTTCTACC |
|  |  | hst5GFP_rev | AGTCGCTCCACGCACTTC |
| *hst6* | Deletion construct | hst6KO5_fwd | GATATCGAATTCCTGCAGCCCGGGGCTACCGTGGGGACAGACAG |
|  |  | hst6KO5_rev | ATGGTGGCCATCTTTCGTTACGAGCATAAGGCAAATG |
|  |  | cbx_hst6KO_fwd | TGCTCGTAACGAAAGATGGCCACCATGGCGT |
|  |  | cbx_hst6KO_rev | GTGATAGTCCGCACTCAGGCCTATTAATGCGGC |
|  |  | hst6KO3_fwd | AATAGGCCTGAGTGCGGACTATCACGTTCTTC |
|  |  | hst6KO3_rev | GGTGGCGGCCGCTCTAGAACTAGTGTAGTCGAGCCATCTGGTG |
|  | Deletion verification | hst6CO5_fwd | CTGCCTGACTCATCATTTGC |
|  |  | cbx1_rev | TCTGGGTTTCGCGAGAGATCTCACAGAGCA |
|  |  | cbx2_fwd | AATTGCACAGATCAAGAAGGACATGGCCGT |
|  |  | hst6CO3_rev | ATTCAAGTTCTCCCAGATGC |
|  |  | hst6KOint_fwd | CAAAGTCTGGCAGTTCTACC |
|  |  | hst6KOint_rev | CTAGCTCTGGAATCGATTCG |
|  | eGFP tagging | hst6ORF_fwd | CGAATTCCTGCAGCCCGGGGGATGTCGACTCTTGCGGCAAACC |
|  |  | hst6ORF_rev | TGCTCACCATAAGGCCAAGCACCTCTGG |
|  |  | eGFPHyg_hst6_fwd | GCTTGGCCTTATGGTGAGCAAGGGCGAG |
|  |  | eGFPHyg_ hst6_rev | CCGCTCAAAGTATTAATGCGGCCGCACAG |
|  |  | hst6Ter_fwd | CGCATTAATACTTTGAGCGGACTATCAC |
|  |  | hst6Ter_rev | CGGCCGCTCTAGAACTAGTGGTCACGACGATGGCAATG |
|  |  | hst6GFP_fwd | GATGTCGACTCTTGCGGCAAACC |
|  |  | hst6GFP_rev | GTCACGACGATGGCAATG |
| Others | RT-qPCR | ppi1_qPCR_fwd | ACATCGTCAAGGCTATCG |
|  |  | ppi1_qPCR_rev | AAAGAACACCGGACTTGG |
|  |  | gapdh_qPCR_fwd | CTTCGGCATTGTTGAGGGTTTG |
|  |  | gapdh_qPCR_rev | TCCTTGGCTGAGGGTCCGTC |
|  |  | sir2_qPCR_fwd | CAAAGTCGCACCTGTATCCGA |
|  |  | sir2_qPCR_rev | AGTGGAGGATTGGCGTCTTGT |
|  |  | eff1-9_qPCR_fwd | CAAGCAAAGAATCCGATCGAGT |
|  |  | ppi1_ChIP_fwd | GGAGGCAAGTCGATCTACGG |
|  |  | ppi1_ChIP_rev | CATGGAAAGAAGACCGGGCT |
|  |  | eff1-9_Pr_ChIP_fwd | ACCTCGCAGCTCAAGGGTAA |
|  |  | eff1-9_Pr_qPCR_rev | AATACCTACCCGCCTGTGAG |
|  |  | eff1-9_orf_ChIP_fwd | AGCAAGCGCGGTGTATGCGA |
|  |  | eff1-9_orf_ChIP_rev | TGCATTTCTCTGACGCTGAG |
|  |  | 06128_Pr_ChIP_fwd | GCCAGGCTGCCAAATAAAAC |
|  |  | 06128_Pr_ChIP_rev | TGGCGCGGAAAAACCAATAC |
|  |  | 06128_orf_ChIP_fwd | TCGCCTGCATCATATTCCAC |
|  |  | 06128_orf_ChIP_rev | TTTCGCGGTTGGAAAACTCG |
|  |  | rsp3_Pr_ChIP_fwd | AGCCTTCTTTTCCACACTGC |
|  |  | rsp3_Pr_ChIP_rev | TGGGGAAACGAGGTTATGAG |
|  |  | rsp3_orf_ChIP_fwd | AGCAAAAGCAAGACGAGCAG |
|  |  | rsp3_orf_ChIP_rev | TCCTTCTTTTGACGCTCCTG |
|  |  | mig2-3_Pr_ChIP_fwd | ATTGTGCGCACATTGCTCTG |
|  |  | mig2-3_Pr_ChIP_rev | TGCTTGAGCTGACTGTATGC |
|  |  | mig2-3_orf_ChIP_fwd | GTTTCCCAGCTTGTTCCTAACG |
|  |  | mig2-3_orf_ChIP_rev | AAAAGCACTGTCCGATAGCG |
|  |  | mig2-6_Pr_ChIP_fwd | TATGATTCTCAGGCGCAGTG |
|  |  | mig2-6_Pr_ChIP_rev | CACCCAAATCTTCCCACATC |
|  |  | mig2-6_orf_ChIP_fwd | AGGATTCGACCATTCTCCAC |
|  |  | mig2-6_orf_ChIP_rev | CAATGTGAACGACAGGCATC |
|  |  | 01241_Pr_ChIP_fwd | TTCTGCCGCATCTAAGTGTG |
|  |  | 01241_Pr_ChIP_rev | AACGGCACAAACAGTACGTC |
|  |  | 01241_orf_ChIP_fwd | ATGTCATGTGGCAGAATCGG |
|  |  | 01241_orf_ChIP_rev | AAGCCAGCCAGGGATTTTTC |
