# Supplementary material for: Systematic characterization of Ustilago maydis sirtuins shows Sir2 as a modulator of pathogenic gene expression
Source: Front Microbiol. 2023 Apr 11;14:1157990. doi: 10.3389/fmicb.2023.1157990 (PMC10126416; doi:10.3389/fmicb.2023.1157990)
Supplement: Supplementary file 3 [file Table_3_v2.docx]

| **Replicate 1** | | | | | | |
| --- | --- | --- | --- | --- | --- | --- |
| **Strain** | **No Tumors** | **Small Tumors** | **Medium Tumors** | **Heavy Tumors** | **Dead Plants** | **n** |
| **WT** | 31 | 37 | 29 | 10 | 5 | 112 |
| **Δ*sir2*** | 17 | 37 | 22 | 13 | 6 | 95 |
| **Replicate 2** | | | | | | |
| **Strain** | **No Tumors** | **Small Tumors** | **Medium Tumors** | **Heavy Tumors** | **Dead Plants** | **n** |
| **WT** | 8 | 13 | 13 | 2 | 2 | 38 |
| **Δ*sir2*** | 3 | 12 | 11 | 6 | 4 | 36 |
| **Replicate 3** | | | | | | |
| **Strain** | **No Tumors** | **Small Tumors** | **Medium Tumors** | **Heavy Tumors** | **Dead Plants** | **n** |
| **WT** | 3 | 6 | 13 | 4 | 3 | 29 |
| **Δ*sir2*** | 3 | 7 | 11 | 4 | 4 | 29 |
|  |  |  |  |  |  |  |
| **Replicate 1 + Replicate 2 + Replicate 3** | | | | | | |
| **Strain** | **No Tumors** | **Small Tumors** | **Medium Tumors** | **Heavy Tumors** | **Dead Plants** | **n** |
| **WT** | 42 | 56 | 55 | 16 | 10 | 179 |
| **Δ*sir2*** | 23 | 56 | 44 | 23 | 14 | 160 |
|  |  |  |  |  |  |  |
| **Percentage** | | | | | |  |
| **Strain** | **No Tumors** | **Small Tumors** | **Medium Tumors** | **Heavy Tumors** | **Dead Plants** |  |
| **WT** | 23,46 | 31,28 | 30,73 | 8,94 | 5,59 |  |
| **Δ*sir2*** | 14,38 | 35,00 | 27,50 | 14,38 | 8,75 |  |

**TABLE S3. Individual infection data of the indicated strains.**

| **Replicate 1** | | | | | | |
| --- | --- | --- | --- | --- | --- | --- |
| **Strain** | **No Tumors** | **Small Tumors** | **Medium Tumors** | **Heavy Tumors** | **Dead Plants** | **n** |
| **WT** | 9 | 3 | 4 | 4 | 1 | 21 |
| **Δ*hst2*** | 8 | 5 | 1 | 8 | 1 | 23 |
| **Replicate 2** | | | | | | |
| **Strain** | **No Tumors** | **Small Tumors** | **Medium Tumors** | **Heavy Tumors** | **Dead Plants** | **n** |
| **WT** | 6 | 7 | 7 | 21 | 5 | 46 |
| **Δ*hst2*** | 12 | 4 | 6 | 20 | 7 | 49 |
|  |  |  |  |  |  |  |
| **Replicate 1 + Replicate 2** | | | | | | |
| **Strain** | **No Tumors** | **Small Tumors** | **Medium Tumors** | **Heavy Tumors** | **Dead Plants** | **n** |
| **WT** | 15 | 10 | 11 | 25 | 6 | 67 |
| **Δ*hst2*** | 20 | 9 | 7 | 28 | 8 | 72 |

|  |  |  |  |  |  |  |
| --- | --- | --- | --- | --- | --- | --- |
| **Percentage** | | | | | |  |
| **Strain** | **No Tumors** | **Small Tumors** | **Medium Tumors** | **Heavy Tumors** | **Dead Plants** |  |
| **WT** | 22,39 | 14,93 | 16,42 | 37,31 | 8,96 |  |
| **Δ*hst2*** | 27,78 | 12,50 | 9,72 | 38,89 | 11,11 |  |

| **Replicate 1** | | | | | | |
| --- | --- | --- | --- | --- | --- | --- |
| **Strain** | **No Tumors** | **Small Tumors** | **Medium Tumors** | **Heavy Tumors** | **Dead Plants** | **n** |
| **WT** | 10 | 3 | 4 | 8 | 2 | 27 |
| **Δ*hst5*** | 9 | 1 | 3 | 8 | 2 | 23 |
| **Δ*hst6*** | 3 | 4 | 4 | 11 | 2 | 24 |
| **Replicate 2** | | | | | | |
| **Strain** | **No Tumors** | **Small Tumors** | **Medium Tumors** | **Heavy Tumors** | **Dead Plants** | **n** |
| **WT** | 6 | 7 | 7 | 21 | 5 | 46 |
| **Δ*hst5*** | 7 | 9 | 6 | 15 | 6 | 43 |
| **Δ*hst6*** | 7 | 10 | 3 | 13 | 8 | 41 |
|  |  |  |  |  |  |  |
| **Replicate 1 + Replicate 2** | | | | | | |
| **Strain** | **No Tumors** | **Small Tumors** | **Medium Tumors** | **Heavy Tumors** | **Dead Plants** | **n** |
| **WT** | 16 | 10 | 11 | 29 | 7 | 73 |
| **Δ*hst5*** | 16 | 10 | 9 | 23 | 8 | 66 |
| **Δ*hst6*** | 10 | 14 | 7 | 24 | 10 | 65 |
|  |  |  |  |  |  |  |
| **Percentage** | | | | | |  |
| **Strain** | **No Tumors** | **Small Tumors** | **Medium Tumors** | **Heavy Tumors** | **Dead Plants** |  |
| **WT** | 21,92 | 13,70 | 15,07 | 39,73 | 9,59 |  |
| **Δ*hst5*** | 24,24 | 15,15 | 13,64 | 34,85 | 12,12 |  |
| **Δ*hst6*** | 15,38 | 21,54 | 10,77 | 36,92 | 15,38 |  |

| **Replicate 1** | | | | | | |
| --- | --- | --- | --- | --- | --- | --- |
| **Strain** | **No tumors** | **Small Tumors** | **Medium Tumors** | **Heavy Tumors** | **Dead Plants** | **n** |
| **WT** | 4 | 4 | 2 | 5 | 4 | 19 |
| **P*pit2*:*sir2* 1c** | 7 | 5 | 2 | 4 | 5 | 23 |
| **Replicate 2** | | | | | | |
| **Strain** | **No tumors** | **Small Tumors** | **Medium Tumors** | **Heavy Tumors** | **Dead Plants** | **n** |
| **WT** | 9 | 5 | 2 | 4 | 2 | 22 |
| **P*pit2*:*sir2* 1c** | 5 | 5 | 3 | 7 | 3 | 23 |
|  |  |  |  |  |  |  |
| **Replicate 1 + Replicate 2** | | | | | | |
| **Strain** | **No Tumors** | **Small Tumors** | **Medium Tumors** | **Heavy Tumors** | **Dead Plants** | **n** |
| **WT** | 13 | 9 | 4 | 9 | 6 | 41 |
| **P*pit2*:*sir2* 1c** | 12 | 10 | 5 | 11 | 8 | 46 |
|  |  |  |  |  |  |  |
| **Percentage** | | | | | |  |
| **Strain** | **No Tumors** | **Small Tumors** | **Medium Tumors** | **Heavy Tumors** | **Dead Plants** |  |
| **WT** | 30,00 | 21,43 | 11,43 | 24,29 | 12,86 |  |
| **P*pit2*:*sir2* 1c** | 26,09 | 21,74 | 10,87 | 23,91 | 17,39 |  |

| **Replicate 1** | | | | | | |
| --- | --- | --- | --- | --- | --- | --- |
| **Strain** | **No tumors** | **Small Tumors** | **Medium Tumors** | **Heavy Tumors** | **Dead Plants** | **n** |
| **WT** | 14 | 11 | 8 | 19 | 1 | 53 |
| **P*pit2*:*sir2* >1c** | 47 | 3 | 0 | 0 | 0 | 50 |
| **Replicate 2** | | | | | | |
| **Strain** | **No tumors** | **Small Tumors** | **Medium Tumors** | **Heavy Tumors** | **Dead Plants** | **n** |
| **WT** | 13 | 3 | 10 | 13 | 1 | 40 |
| **P*pit2*:*sir2* >1c** | 36 | 14 | 0 | 0 | 0 | 50 |
| **Replicate 3** | | | | | | |
| **Strain** | **No tumors** | **Small Tumors** | **Medium Tumors** | **Heavy Tumors** | **Dead Plants** | **n** |
| **WT** | 12 | 7 | 4 | 9 | 0 | 32 |
| **P*pit2*:*sir2* >1c** | 22 | 12 | 0 | 0 | 0 | 34 |
|  |  |  |  |  |  |  |
| **Replicate 1 + Replicate 2 + Replicate 3** | | | | | | |
| **Strain** | **No Tumors** | **Small Tumors** | **Medium Tumors** | **Heavy Tumors** | **Dead Plants** | **n** |
| **WT** | 39 | 21 | 22 | 41 | 2 | 125 |
| **P*pit2*:*sir2* >1c** | 105 | 29 | 0 | 0 | 0 | 134 |
|  |  |  |  |  |  |  |
| **Percentage** | | | | | |  |
| **Strain** | **No Tumors** | **Small Tumors** | **Medium Tumors** | **Heavy Tumors** | **Dead Plants** |  |
| **WT** | 31,20 | 16,80 | 17,60 | 32,80 | 1,60 |  |
| **P*pit2*:*sir2* >1c** | 78,36 | 21,64 | 0,00 | 0,00 | 0,00 |  |
