# Supplementary material for: Systematic characterization of Ustilago maydis sirtuins shows Sir2 as a modulator of pathogenic gene expression
Source: Front Microbiol. 2023 Apr 11;14:1157990. doi: 10.3389/fmicb.2023.1157990 (PMC10126416; doi:10.3389/fmicb.2023.1157990)
Supplement: Supplementary file 9 [file Data_Sheet_1_v1.pdf]

Supplementary Figure S1

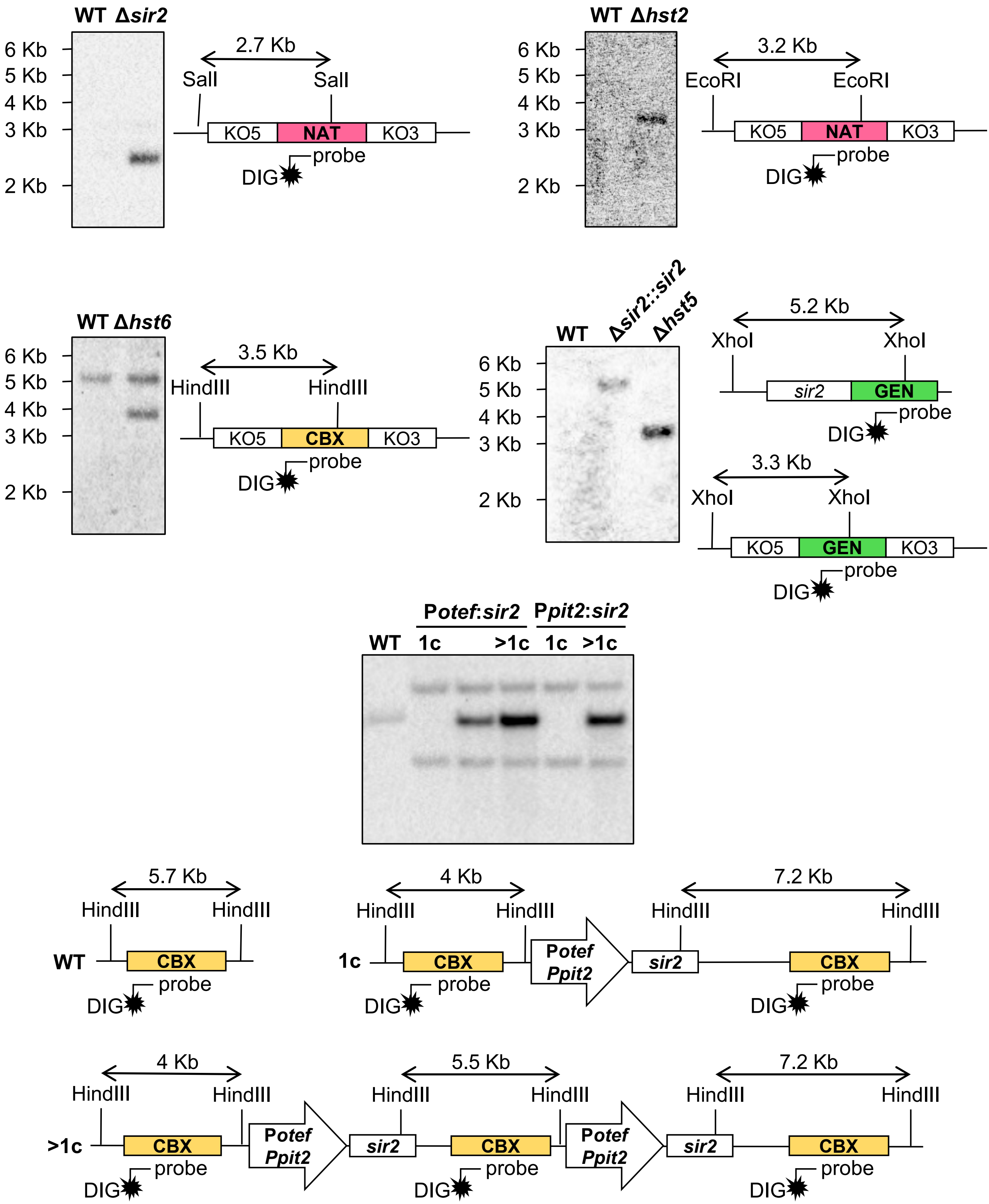

SUPPLEMENTARY FIGURE S1. Verification by Southern Blot of the strains done in this work is shown. Schematic representation of the construction and the size of the expected band are represented for each strain.

Supplementary Figure S2

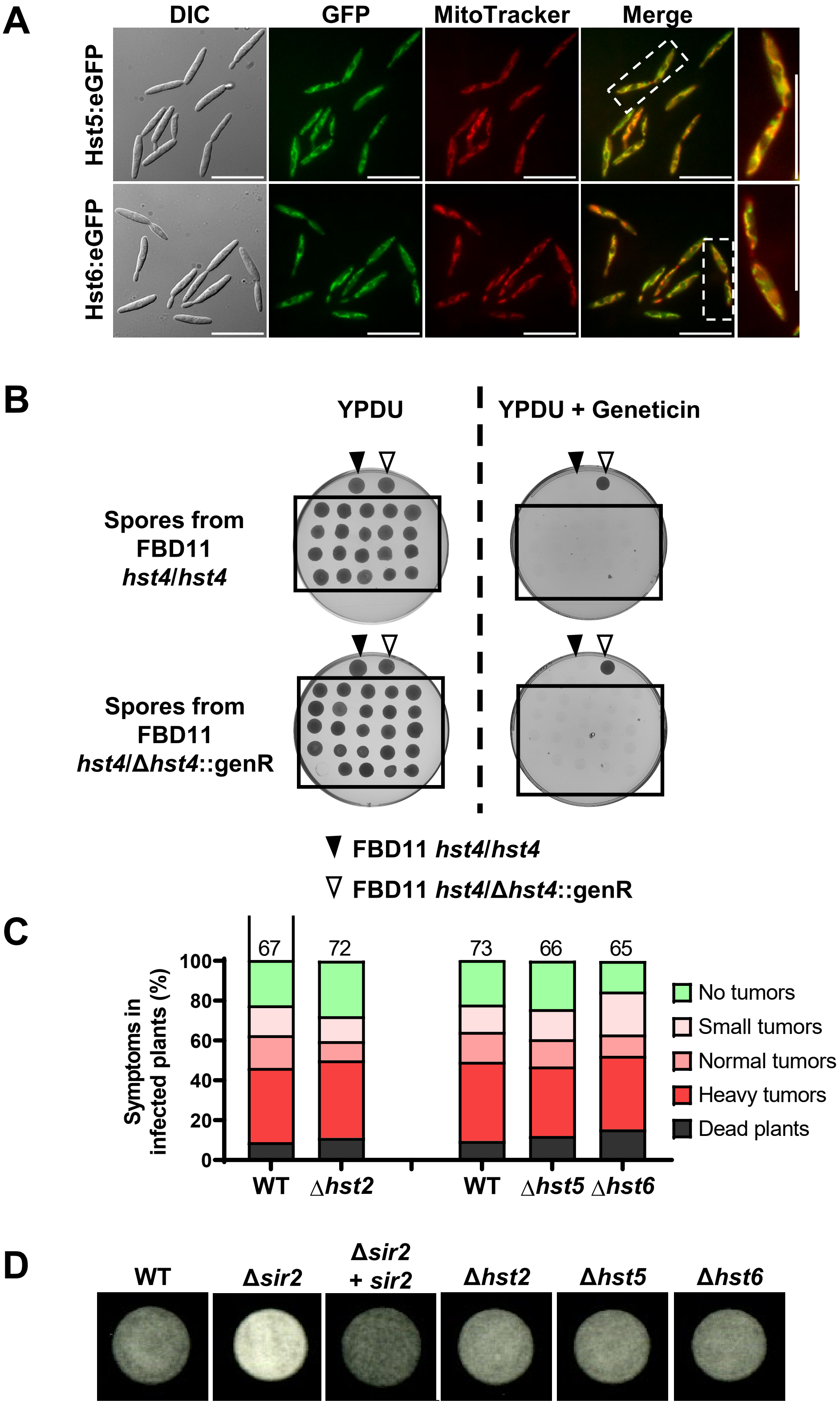

SUPPLEMENTARY FIGURE S2. Hst5 and Hst6 localize in the mitochondria, deletion of *hst4* is not viable for *Ustilago maydis* and Hst2, Hst5 and Hst6 have no effects in the virulence of the fungus. **(A)** Confocal microscopy of *U. maydis* strains expressing the indicated sirtuins tagged with eGFP in its own loci. Mitochondria were stained with MitoTracker. Scale bar represents 20 µm. **(B)** FBD11 wild-type and FBD11 *hst4*/Δ*hst4*::genR diploid strains were inoculated on maize plant. None of the singularized colonies obtained from germinated spores were able to grow on selective medium (YPDU plus geneticin). YPDU plates were used as control. Control strains were spotted on top of the plates. **(C)** Quantification of symptoms for plants infected with the indicated strains at 14 dpi. Total number of infected plants is indicated above each column. Two biological replicates were analyzed. Mann–Whitney statistical test was performed (ns, no significant; **** p-value < 0.001). **(D)** Filamentation of wild-type and the indicated sirtuins mutants grown on PD-charcoal plates for 18 hours at 25ºC.

Supplementary Figure S3

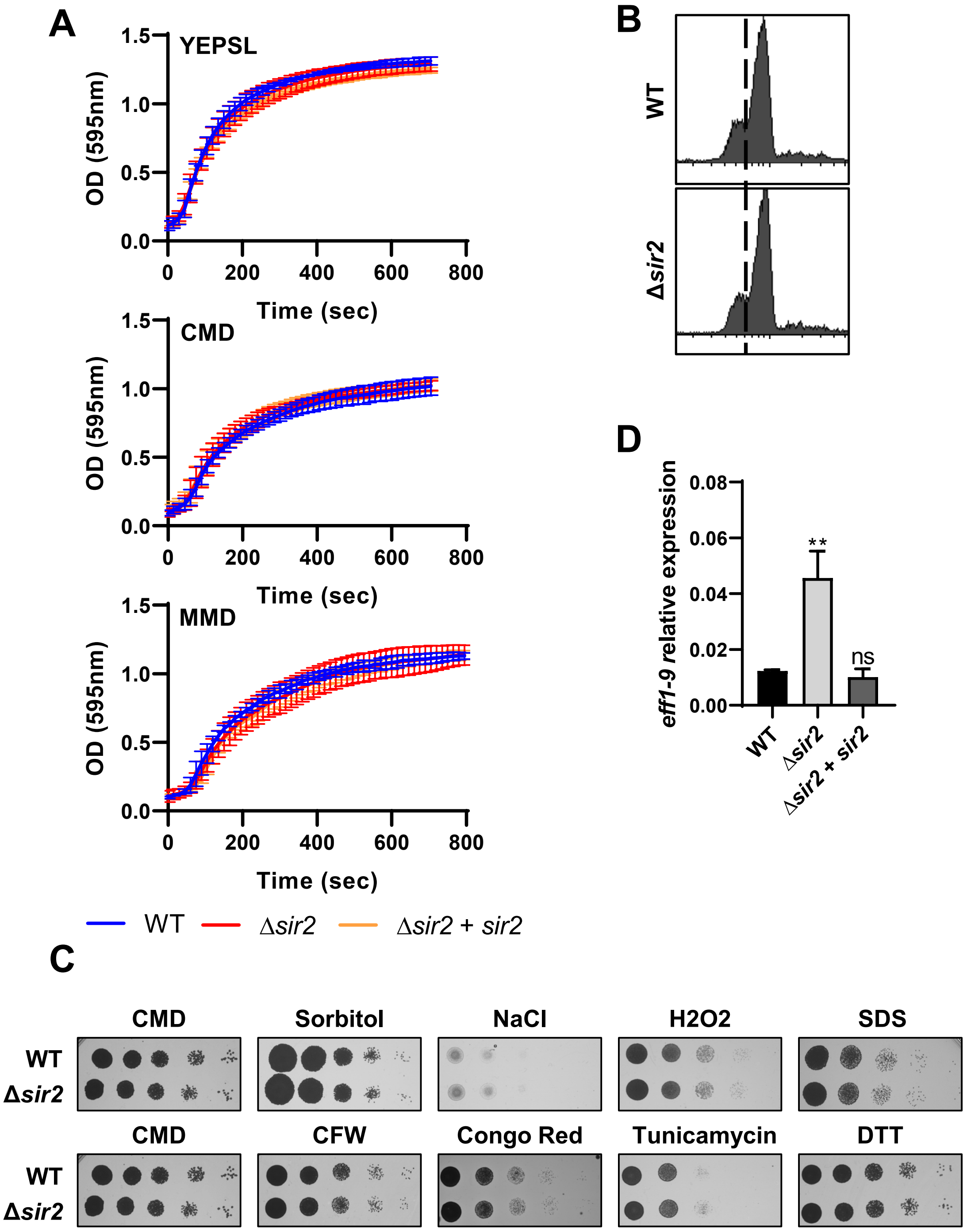

SUPPLEMENTARY FIGURE S3. Deletion of *sir2* has not pleiotropic effects and is complemented by reintroducing *sir2* gene. **(A)** Growth curve of wild-type and Δ*sir2* mutant growing in YEPSL, CMD or MMD media. Error bars represent the standard deviation from three independent replicates. **(B)** Flow cytometry analysis of the DNA content of wild-type and Δ*sir2* mutant grown in CMD medium. Relative fluorescence intensities are given on horizontal axes and vertical axes reflect cell numbers. **(C)** Spot tests to assay osmotic stress (sorbitol and NaCl), oxidative stress (H2O2), membrane integrity (SDS), cell wall integrity (calcofluor white (CFW) and Congo red) and endoplasmic reticulum stress (Tunicamycine and DTT). CMD without drug was used as growth control. **(D)** *eff1-9* expression in axenic culture of wild-type, Δ*sir2* and the Δ*sir2* complementation strains measured by RT-qPCR. U. maydis *ppi1* gene was used for normalization. Error bars represent the standard deviation from three independent replicates. Student's t-test statistical analysis was performed (ns, not significant; ** $p\text{-value} < 0.005$).

Supplementary Figure S4

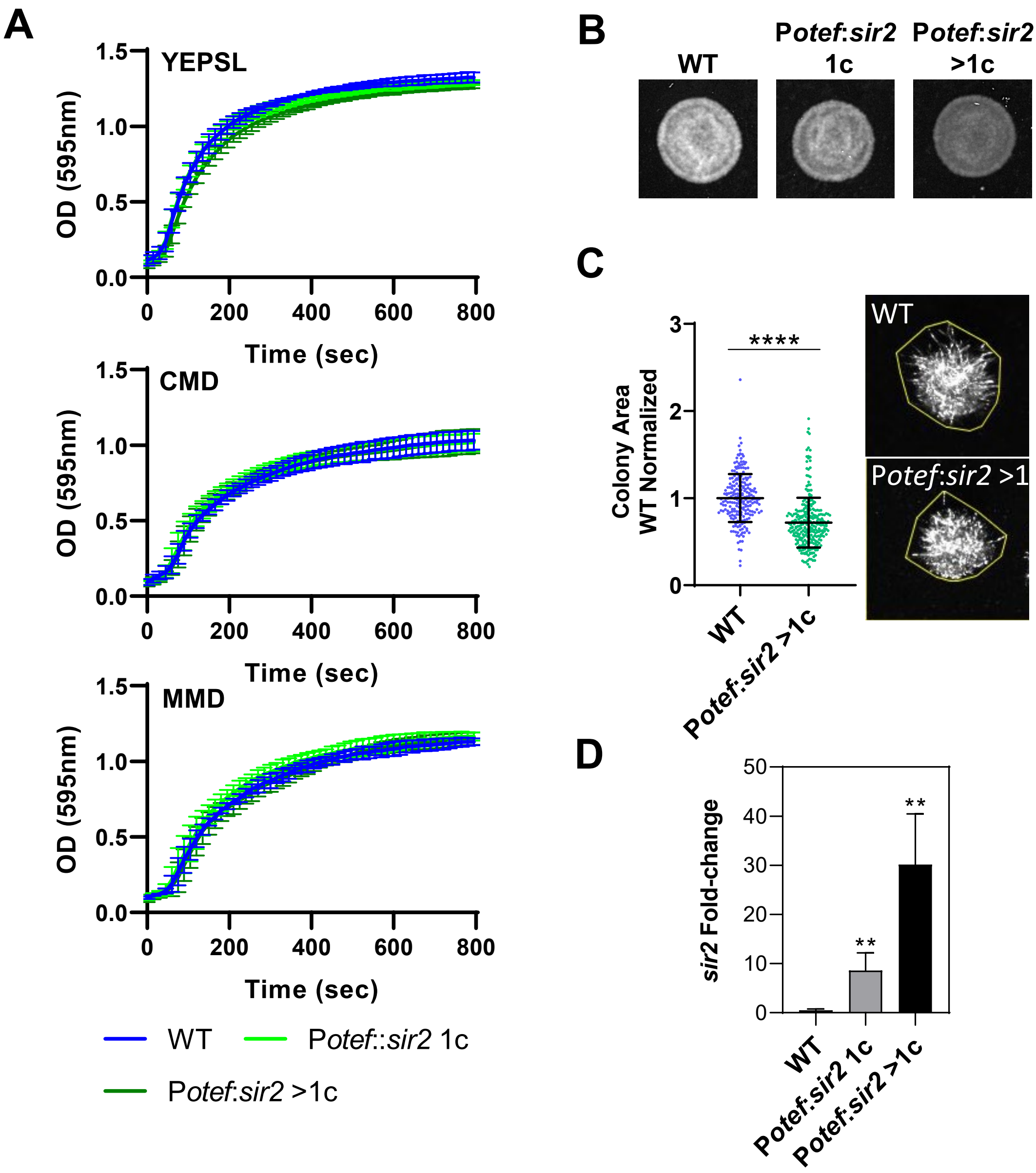

SUPPLEMENTARY FIGURE S4. Sir2 overexpression does not affect cell growth but reduces the filament formation on PD-charcoal plates. **(A)** Growth curve of wild-type, P*otef*:*sir2* 1c and P*otef*:*sir2* >1c mutants growing in YEPSL, CMD or MMD media. Error bars represent the standard deviation from three independent replicates. **(B)** Filamentation of wild-type and *sir2* overexpression mutants containing one (1c) or more copies (>1c) of the P*otef*:*sir2* construct, grown on PD–charcoal plates for 18 hours at 25ºC. **(C)** Quantification of the area of the wild-type and Δ*sir2* mutant single colonies grown on PD–charcoal plates for 48 hours at 25ºC. The colony area was measured as indicated in the stereoscopic images. Data was normalized with the mean of the area of the wild-type colonies. Three biological replicates were analyzed. Student's t-test statistical analysis was performed (**** p-value < 0.001). **(D)** *sir2* expression levels in axenic culture of wild-type and the *sir2* overexpression mutants measured by RT-qPCR. *U. maydis ppi1* was used as reference gene. Values were normalized to wild-type. Error bars represent the standard deviation from at least three independent replicates. Student's t-test statistical analysis was performed (** p-value < 0.005).

Supplementary Figure S5

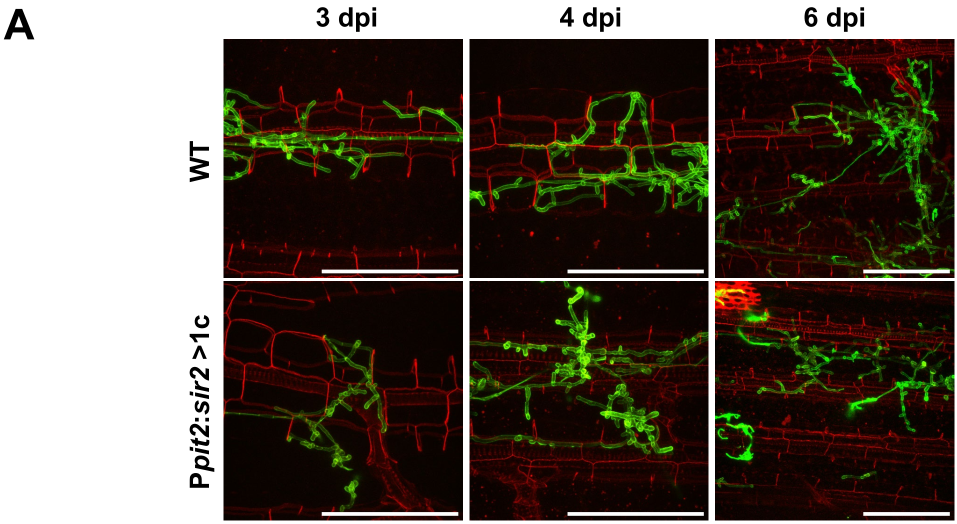

SUPPLEMENTARY FIGURE S5. Morphology of *U. maydis* inside the plant is not affected with the overexpression of *sir2*. Maize leaves from plants infected with wild-type and the P*pit2*:*sir2* >1c mutant at 3, 4 and 6 dpi were stained with propidium iodide (red) and *U. maydis* hyphae with WGA-AF-488 (green) and visualized by fluorescence microscopy. Scale bar represents 100 µm.

Supplementary Method S1. Growth curve and stress assay.

For the growth curve assay, U. maydis cells were grown to the exponential phase and diluted to OD600 of 0.1 in YEPSL, CM supplemented with 1% D-glucose (CMD) or MM supplemented with 1% D-glucose (MMD) (Holliday, 1974). Cell growth at 28ºC with continuous shaking was analyzed over 24 h using a Spark 10M fluorescence microplate reader (Tecan, Männedorf, Switzerland). Cell wall integrity, membrane integrity, osmotic, endoplasmic reticulum and oxidative stress assays were carried out with cultures grown at 28º C to the exponential phase in CMD and spotted at 0.4 OD600 onto CMD plates supplemented with calcofluor white (CFW) 40 µg/mL (Sigma-Aldrich, Darmstadt, Germany), Congo Red 50 µg/mL (Sigma-Aldrich, St. Louis, MO, USA), 4 mM DTT (iNtRON Biotechnology, Seongnam, Gyeonggi, ROK), tunicamycin 1 µg/mL (Sigma-Aldrich, Darmstadt, Germany), sorbitol 1 M (Sigma-Aldrich, Darmstadt, Germany), 2% DMSO (Sigma-Aldrich, Darmstadt, Germany), H2O2 0.75 mM (Sigma-Aldrich, Darmstadt, Germany), NaCl 1 M (Sigma-Aldrich, Darmstadt, Germany) and 0.005% SDS (Sigma-Aldrich, Darmstadt, Germany). Plates were incubated at 28º C for 48 h.

Supplementary Method S2. Spore germination.

Spore germination was conducted according to published protocols (Eichhorn et al., 2006). Mature tumors of plants infected with U. maydis FBD11 strain and it derivate FBD11 Δhst4 were harvested 21 dpi and dried at 37ºC for 2 days. Spores were rehydrated with distilled water, crushed with a mortar and incubate with 3% CuSO4 solution at RT for 4 hours. Washed spores were germinated in YPDU plated supplemented with Ampicillin (100 µg/ml), Chloramphenicol (25 µg/mL) and Tetracycline (10 µg/ml). To test FBD11 Δhst4 viability, singularized colonies from germinated spores were grown in YPDU supplemented with geneticin (2 µg/ml).

Supplementary Method S3. Flow cytometry.

To analyze the Δsir2 mutant DNA content, we followed the previously described protocol (García-Muse et al., 2003). Cells were grown in CMD medium to exponential phase. Prior to analysis, cells were harvested, washed twice with cold water, fixed in 70% ethanol overnight, and resuspended in 50 mM sodium citrate, pH 7.5. Cellular RNA was eliminated by incubation with RNase A (0.25 mg/mL) at 50ºC for 1 hour and then cells were incubated with proteinase K (1 mg/mL) for 1 hour at 50ºC. Cells were stained with propidium iodide (16 mg/mL) and the fluorescence of 10.000 cells was measured using a FACSCalibur flow cytometer (Becton Dickinson, East Rutherford, NJ, USA) with a 530/30 bandpass filter.

Supplementary Method References

Eichhorn, H., Lessing, F., Winterberg, B., Schirawski, J., Kämper, J., Müller, P., et al. (2006). A ferroxidation/permeation iron uptake system is required for virulence in *Ustilago maydis*. *Plant Cell* 18, 3332–3345. doi: 10.1105/tpc.106.043588.

García-Muse, T., Steinberg, G., and Pérez-Martín, J. (2003). Pheromone-induced G2 arrest in the phytopathogenic fungus *Ustilago maydis*. *Eukaryot. Cell* 2, 494–500. doi: 10.1128/EC.2.3.494-500.2003.

Holliday, R. (1974). "*Ustilago maydis*," in *Bacteria, Bacteriophages, and Fungi* (Boston, MA: Springer US), 575–595. doi: 10.1007/978-1-4899-1710-2_31.
